# Risk prediction models for postoperative atrial fibrillation in patients with lung cancer: a systematic review and meta-analysis

**DOI:** 10.3389/fcvm.2026.1779009

**Published:** 2026-06-02

**Authors:** Fei Yang, Tenglu Sun, Yi Shang, Yuanyuan Chen, Jinxiang Wu, Xuli Shang

**Affiliations:** 1 Medicine School of Lishui University, Lishui, Zhejiang, China; 2 Changchun Humanities and Sciences College, Changchun, China; 3 Department of Otorhinolaryngology, Lishui Hospital of Wenzhou Medical University, The First Affiliated Hospital of Lishui University, Lishui People's Hospital, Lishui, Zhejiang, China; 4 Department of Hepatology and Infectious Diseases, Lishui Hospital of Wenzhou Medical University, The First Affiliated Hospital of Lishui University, Lishui People's Hospital, Lishui, Zhejiang, China; 5 Department of Nursing, Lishui Hospital of Wenzhou Medical University, The First Affiliated Hospital of Lishui University, Lishui People's Hospital, Lishui, Zhejiang, China

**Keywords:** lung cancer, meta-analysis, postoperative atrial fibrillation, risk prediction model, systematic review

## Abstract

**Background:**

Postoperative atrial fibrillation (POAF) is a common and clinically significant complication following lung cancer surgery, associated with increased morbidity and mortality. Although numerous prediction models have been developed to estimate POAF risk, their overall performance and methodological quality remain unclear.

**Methods:**

A systematic review and meta-analysis were conducted in accordance with the PRISMA 2020 guidelines, and the protocol was registered with PROSPERO (CRD42025115874). Chinese and English databases were searched from their inception until 30 May 2024. Studies that developed or validated prediction models for postoperative atrial fibrillation (POAF) in patients with surgically treated lung cancer were included. Data were extracted using the CHARMS checklist and the risk of bias was assessed using PROBAST. A random-effects meta-analysis was performed to pool the discriminative performance of the eligible models, using the area under the curve (AUC).

**Results:**

Six studies were included. Most models were developed using logistic regression, with age, sex, cardiovascular comorbidities and surgical factors being the most common predictors. Reported area under the curve (AUC) values ranged from 0.72 to 0.89. The pooled AUC was 0.79 (95% CI: 0.71–0.87), which indicates good overall discrimination. However, substantial heterogeneity was observed (*I*^2^ = 98.7%). Subgroup analysis with consistent outcome definitions showed reduced heterogeneity. All studies were judged to have a high overall risk of bias.

**Conclusions:**

Current POAF prediction models for lung cancer patients show acceptable discriminative ability but are limited by methodological weaknesses and lack of external validation, restricting their clinical applicability.

**Systematic Review Registration:**

https://www.crd.york.ac.uk/PROSPERO/view/CRD420251158742, identifier CRD420251158742.

## Background

Lung cancer remains one of the most prevalent and lethal malignancies worldwide, with a concerning upward trend in recent years (1). Current data indicate an annual incidence of approximately 2.5 million cases and 1.8 million deaths, accounting for 18.7% of all cancer-related mortality (2). Despite significant advancements in early detection and therapeutic modalities, the clinical manifestations of lung cancer are often insidious, frequently leading to delayed diagnoses (3). Consequently, a substantial proportion of patients are diagnosed at advanced stages, which limits treatment efficacy and contributes to persistently high mortality rates (4, 5). For patients undergoing surgical intervention, long-term survival is intricately linked to the occurrence of postoperative complications, which serve as a primary driver of mortality (6). Statistics suggest that approximately 30% of lung cancer patients experience complications following surgery, with a postoperative mortality rate of around 5% (7, 8). While surgical techniques have evolved—particularly with the advent of minimally invasive procedures—postoperative complications, especially cardiovascular events, remain a formidable challenge (9). Among these, atrial fibrillation (AF) is a predominant cardiovascular complication that significantly elevates the risk of postoperative death, necessitating robust risk assessment and preventive strategies (10).

Postoperative atrial fibrillation (POAF) is the most frequent cardiovascular complication following lung cancer surgery. Its incidence exhibits marked variability, typically ranging from 5% to 20%, but can exceed 20% in high-risk populations or following extensive procedures such as pneumonectomy (11). Furthermore, the development of POAF is closely associated with pre-existing comorbidities, including hypertension and coronary artery disease (12), highlighting the urgent need for timely intervention in susceptible individuals.

Beyond extending hospital stays, POAF can precipitate severe adverse events, such as heart failure, thromboembolism, and stroke, all of which profoundly impact patient prognosis and quality of life (10). Research has indicated that patients suffering from lung cancer who subsequently develop postoperative atrial fibrillation have a considerably lower long-term survival probability. The reported 3-year survival rate for this group was 62.9%, falling to 52.0% at 5 years. In comparison, patients without this complication have reported 82.4% and 72.7% survival rates at the same points in time (13).The associated risk of thromboembolic events and cardiac dysfunction further exacerbates the patient's condition, leading to higher recurrence rates and complicating subsequent therapeutic decisions (10). Therefore, early identification and proactive management of POAF are paramount to improving clinical outcomes.

Prognostic models are mathematical equations incorporating multiple variables. They are utilised to estimate the probability that an individual in a specific health state will experience a particular health outcome (14). In this context, the development of models capable of accurately predicting the risk of POAF in patients with lung cancer has attracted considerable interest. This is due to the fact that POAF is a common and serious complication that occurs following thoracic surgery. The establishment of risk models for patients with lung cancer has been identified as a solution to numerous clinical and research problems. However, studies have found that the risk of POAF is differentially distributed among different surgical approaches (15) and patient comorbidity profiles (16). Prediction models have the capacity to assist healthcare providers in optimising their decisions. This includes the accurate and rapid identification of patients who require targeted preventive measures and more intensive postoperative monitoring. Moreover, these models have the potential to inform patients with lung cancer and their family members of the risk of POAF, thereby enhancing their awareness and facilitating shared decision-making and compliance with preventive strategies (17).

In recent years, a growing body of research has been undertaken with the aim of developing and validating risk prediction models for the occurrence of postoperative atrial fibrillation in patients diagnosed with lung cancer (18, 19). Nevertheless, a widely accepted and authoritative prediction model that has been endorsed by guidelines has yet to be developed. Furthermore, these models have been developed using small cohorts and lack external validation. Moreover, they have rarely been applied in clinical practice, and the methodological quality has rarely been thoroughly and critically assessed (20). New models have been published; however, this problem remains unresolved. The present systematic review aimed to screen and systematically review published studies on existing risk prediction models for postoperative atrial fibrillation in patients with lung cancer.

## Methods

### Study design

The present systematic review was conducted in accordance with the Preferred Reporting Items for Systematic Reviews and Meta-Analyses (PRISMA) 2020 guidelines. The study protocol was duly registered in PROSPERO (https://www.crd.york.ac.uk/PROSPERO/view/CRD420251158742).

### Search strategy

**Figure 1:**
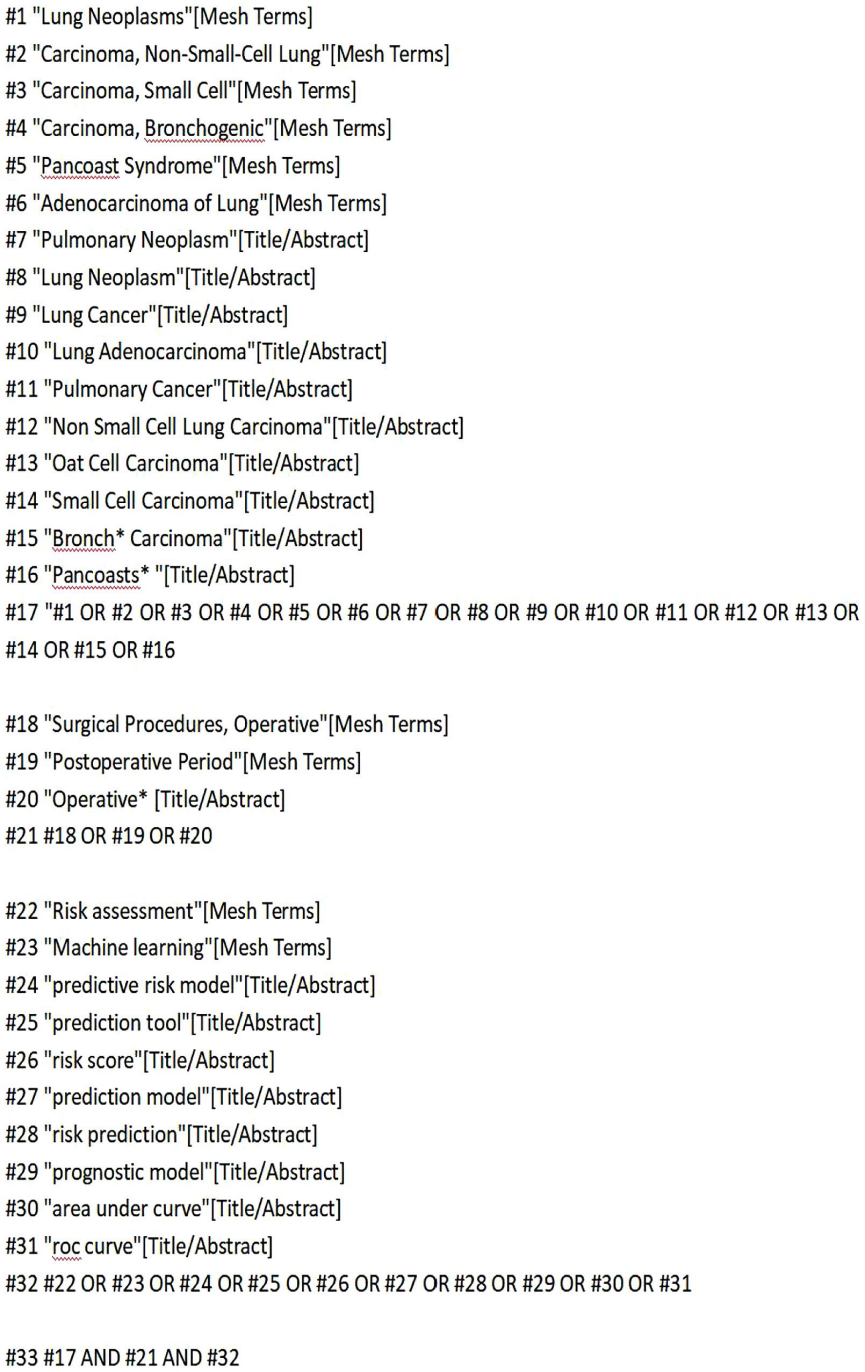
Search strategy.

A comprehensive search was conducted using both Chinese and English databases, with consideration given to the significant population size and linguistic universality of the two languages. The Wan fang, China National Knowledge Infrastructure (CNKI), China Science and Technology Journal (VIP), PubMed, Cochrane Library, Web of Science, Embase and Cumulative Index to Nursing and Allied Health Literature (CINAHL) databases were searched from inception until May 30, 2024, using the following keywords: “Lung Neoplasms,” “Carcinoma, Non-Small-Cell Lung,” “Carcinoma, Small Cell,” “Carcinoma, Bronchogenic,” “Pancoast Syndrome,” “Adenocarcinoma of Lung,” “Adenocarcinoma of Lung,” “Lung Cancer,” “Oat Cell Carcinoma,” “Surgical Procedures, Operative,” “Postoperative Period,” “Atrial Fibrillation,” “Atrial Flutter,” “Risk prediction model,” “Risk factor,” “Predictor,” “Model,” and “Risk Score.” The retrieval method, using PubMed as an example, is shown in Figure 1. Additional relevant studies were identified by reviewing the reference lists of the retrieved studies and review articles.

The systematic review was conducted utilising the PICOTS system, as recommended by the Critical Appraisal and Data Extraction for Systematic Reviews of Prediction Modelling Studies (CHARMS) checklist (21). The system has been developed for the purpose of assisting in the formulation of the review's objective, search strategy, and study inclusion and exclusion criteria (22). The key items of our systematic review are described below:

P (Population): Patients diagnosed with lung cancer who are undergoing surgical resection.
I (Intervention model): The following study will examine risk prediction models for postoperative atrial fibrillation in patients with lung cancer that were developed and published (prediction score ≥ 2).
C (Comparator): No competing model.
O (Outcome): The outcome focused on POAF, rather than its subgroups or other postoperative complications.
T (Timing): The postoperative outcomes were predicted following a comprehensive evaluation of the patients' preoperative baseline characteristics, clinical assessment data, and laboratory indicators obtained before or at the time of surgery.
S (Setting): The primary objective of the risk prediction models was to personalise the prediction of postoperative atrial fibrillation risk in patients with lung cancer, thereby facilitating the implementation of preventive measures for this adverse event.

### Inclusion and exclusion criteria

The inclusion criteria for studies were as follows: (1) inclusion of patients with lung cancer, (2) use of observational study design, (3) report of a prediction model, and (4) postoperative atrial fibrillation as the outcome of interest. The exclusion criteria were as follows: (1) no development of a prediction model, (2) outcomes limited to atrial fibrillation subtypes, (3) not written in English or Chinese, and (4) full text not retrievable despite contacting authors via email.

### Study selection and screening

The screening process was conducted independently by two authors (FY and TS). Initially, duplicate studies were removed, and the remaining studies were assessed based on their titles and abstracts to determine their eligibility. Following the application of the inclusion and exclusion criteria, a full-text review was conducted, and the reference lists of all eligible studies were examined to identify any potentially relevant studies. In instances of discordance pertaining to the selection of studies, a deliberative process involving three authors (FY, TS and YC) was initiated to achieve a consensus.

### Data extraction

The evaluation process involved a dual-reviewer approach, with each reviewer independently assessing the search results. The eligibility of the full-text reports was assessed, and any discrepancies were resolved through discussion or by a third reviewer. The data from the articles selected for inclusion in the final review were extracted using the Critical Appraisal and Data Extraction for Systematic Reviews of Prediction Modeling Studies (CHARMS) checklist. The information extracted from the selected studies was then categorised into two groups: (1) Basic information, including details on the authors, year of publication, research design, participants, outcome indicator, observation time, and atrial fibrillation rate; and (2) Model information, including information related to the prediction model, such as model development method, model validation type, variable selection method, predictive factors, model performance, calibration method, and model presentation.

### Quality assessment

The risk of bias and the applicability of the included studies were assessed using the Prediction Model Risk of Bias Assessment Tool (PROBAST) checklist. The presence of bias and concerns regarding the applicability of the studies was evaluated independently by two authors (FY and TS). The PROBAST checklist is utilised for the critical appraisal of studies on developing, validating, or updating prediction models for individualised predictions. It comprises 20 signaling questions categorized into four domains: participants, predictors, outcomes, and analysis. Each signaling question can be answered as “yes,” “probably yes,” “no,” “probably no,” or “no information.” If at least one signaling question in a domain is answeredas “no” or “probably no,” that domain would be considered at high risk of bias. The overall risk of bias is considered low only when all domains are judged to have a low risk of bias.

### Data analysis

The analysis was conducted utilising the Review Manager and R Studio software. The heterogeneity of the included studies was assessed using the *I*^2^ index and Cochrane's *Q* test. The *I*^2^ index is a measure of heterogeneity. Values of 25%, 50%, and 75% are indicative of low, moderate, and high levels of heterogeneity, respectively (23). The employment of random- or fixed-effects models was contingent upon the heterogeneity of the analysis results. A sensitivity analysis was conducted utilising the leave-one-out method.

## Results

### Study selection

**Figure 2:**
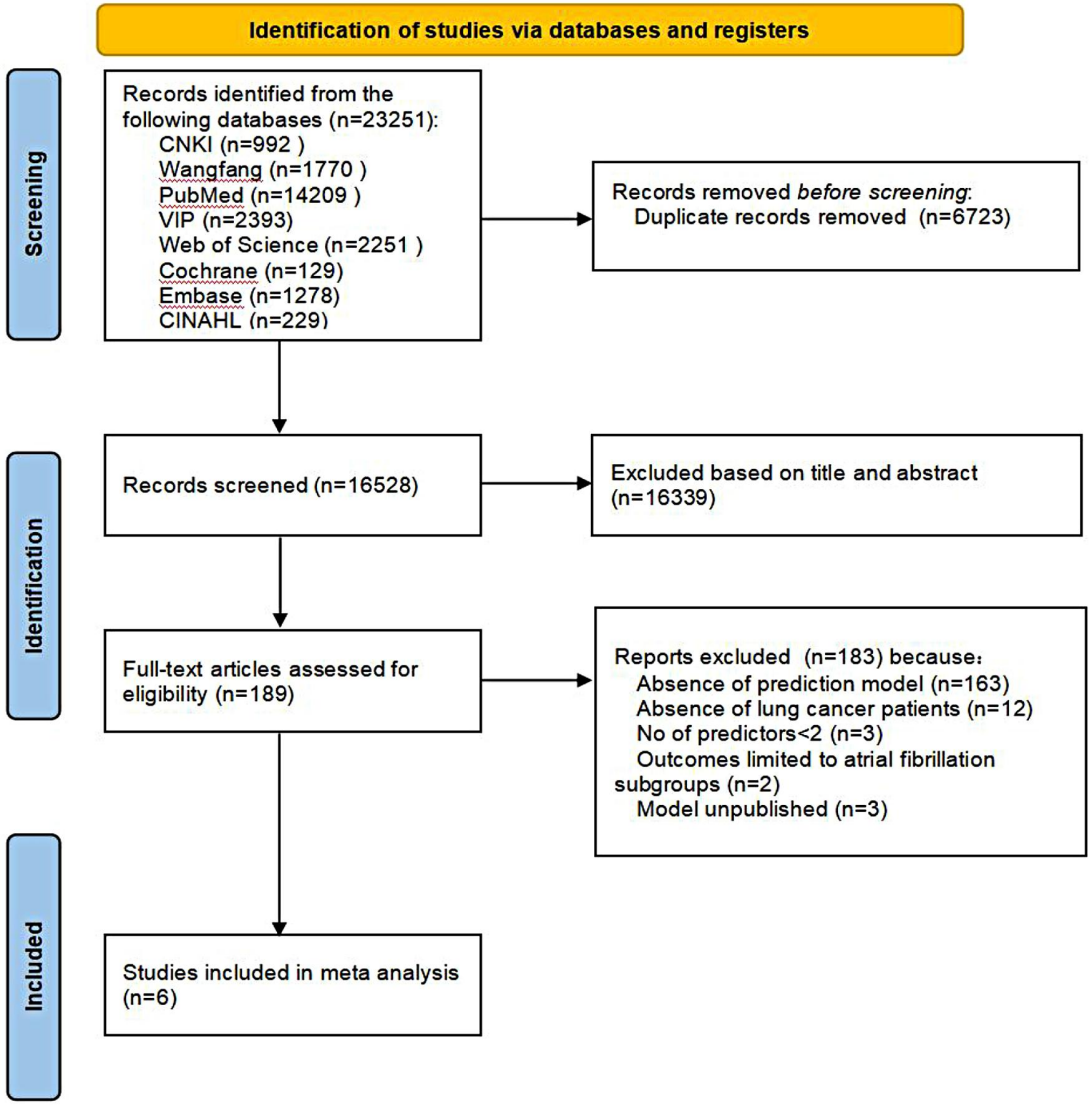
PRISMA flow diagram of the literature search and selection process.

The PRISMA 2020 guideline flowchart is shown in Figure 2. The preliminary database search yielded 23,251 references. Following the removal of duplicates, a total of 16,528 records were retained across all databases. A total of 189 titles and abstracts were reviewed. Following the application of the predetermined eligibility criteria, 183 studies were excluded from the analysis. The specific reasons for exclusion included prediction models not established in 163 studies, inconsistent study populations in 12 studies, and studies containing fewer than two predictors in 3 study, outcomes limited to atrial fibrillation subgroups in 2. The final analysis included 6 studies.

### Study characteristics

Table 1 presents a summary of the characteristics of the six studies that were included in the analysis. The studies were published between 2010 and 2025. Five of the studies were conducted in China, while one were conducted in the United States. Furthermore, five of the studies were retrospective in design, while one was a prospective cross-sectional study. Five studies were conducted across multiple centers, and one underwent single center validation. The sample sizes ranged from 17 to 58,899 participants across the studies.

**Table 1:** Overview of basic data of the included studies.

Author (Year)	Country	Participants	Study design	Data source	Type of surgery	Outcome indicator observation time	Main outcome	Atrial fibrillation rate (%)
Onaitis et al. (24)	USA	13,906 patients with lobe/total lung resection of lung cancer from 2002 to 2008.	Retrospective study	STS	lobectomy and pneumonectomy	Duration of hospital stay	POAF	(1,755/13,906) 12.6
Zhang (26)	China	1,201 lung cancer patients who met the exclusion criteria from the thoracic surgery department of a tertiary hospital in Dalian from January to October 2021.	Prospective cross-sectional study	One hospital	/	Within 72 h postoperatively	POAF	(78/1,201) 6.5
Zhanga et al. (25)	China	184 lung cancer patients aged 60 years or older who underwent thoracoscopic resection between 2022 and 2023.	Retrospective study	One hospital	Thoracoscopic resection	48 h after surgery	POAF	(17/184) 9.2
Chen et al. (19)	China	A: 58,899 patients with lung resection in 40 cohort studies; B: 1,546 patients with lung resection for lung cancer who were aged ≥18 years between 2015 and 2017.	Retrospective study	A: 40 published studies B: One hospital	/	7-day admission	POAF	A: (4,734/58,899) 8 B: (60/1,546)3.9
Niu (27)	China	582 patients who met the criteria from the thoracic surgery department of a tertiary hospital from January 2017 to October 2021.	Retrospective study	One hospital	/	From after the operation to the day after the drainage tube is removed	POAF	A: (80/582) 13.75
Tong et al. (18)	China	5,419 consecutive patients who underwent thoracoscopic anatomical lung cancer surgery from the thoracic surgery department of a tertiary hospital from January 2016 to December 2018.	Retrospective study	Two hospitals	Thoracoscopic anatomical lung cancer surgery	Duration of hospital stay	POAF	A: (230/8,717) 2.6 B: (29/2,941) 1

STS, the society of thoracic surgeons; POAF, postoperative atrial fibrillation; A, development cohort; B, validation cohort; “/”, not reported.

Table 2 presents detailed information on the models utilised in the six studies. Logistic regression was employed for model development in five studies, while meta-analysis combined with risk score assignment was used for model development in one study. The most frequently employed predictors were age and gender, which were utilised in five studies. In three studies, other frequently used predictors included surgical characteristics such as the procedure type or its extent. Underlying diseases of the cardiovascular system were used in there studies.

**Table 2:** Overview of the information of the included prediction models.

Author (Year)	Missing data handling	Continuous variable processing method	Variable selection	Model development method	Calibration method	Validation method	Predictive factor	Model performance	Model presentation
Onaitis et al. (24)	Multiple imputation	Continuous variable	a reduced model	Multivariate logistic regression models	Hosmer-Lemeshow and Calibration plot	Internal validation	Age, Extent of operation, Male sex, Race, Clinical stage.	B:0.664	List the strong predictors and Display the calibration curve
Zhang (26)	/	Categorical variables	Multivariate analysis after univariate screening	Multivariate logistic regression model	ROC curve and Calibration plot	Internal validation	Gender, Age ≥ 60 years, history of cardiovascular disease, scope of surgery.	A:0.722 (0.647–0.798) sensitivity:0.755 specificity:0.618 B:0.742 (0.664–0.821)	Risk score formula derived from the OR values of each factor
Zhang et al. (25)	/	Categorical variables	Multivariate analysis after univariate screening	Multivariate logistic regression model	Hosmer-Lemeshow and Calibration plot	Internal validation	Male gender, history of cardiovascular disease, low postoperative oxygen partial pressure, preoperative pulmonary infection.	A:0.854 sensitivity:0.826 specificity:0.815 C:0.801	Nomogram model
Chen et al. (19)	/	Categorical variables	sensitivity or subgroup analyses	Convert relative risk into risk scores	ROC curve and Calibration plot	External validation	Age (≥70 years), Gender, COPD, CAD, Heart failure, Extent of surgery Surgical approach.	B:0.89	Calculate the total score as the risk score based on the RR value of each factor
Niu (27)	Complete case analysis	Categorical variables	Multivariate analysis after univariate screening	Multivariate logistic regression model	Hosmer-Lemeshow、ROC curve and Calibration plot	Internal validation	Advanced age (≥65 years), Gender, Preoperative hypokalemia, Preoperative hypohemoglobin, Preoperative thyroid dysfunction, Thoracotomy, lobectomy, dissection of group 5.6 lymph nodes.	A:0.890 (0.845−0.934) sensitivity:0.788 specificity:0.877 B:0.888 (0.887-0.890) sensitivity:0.879 specificity:0.785	Nomogram model
Tong et al. (18)	Complete case analysis	Continuous variable	Multivariate analysis after univariate screening	Multivariable logistic regression model	Hosmer-Lemeshow test and Brier score and ROC curve	Internal validation and External validation	Age, hypertension, preoperative treatment, clinical tumor stage, intraoperative, arrhythmia, transfusion, operative time.	A:0.740 (0.709–0.771) sensitivity:0.826 specificity:0.514 B:0.768	Nomogram model

COPD, chronic obstructive pulmonary disease; CAD, coronary artery disease; A, development cohort; B, validation cohort; C, C-index.

Six studies reported the performance of the model. The area under the receiver operating characteristic curve (AUC) or C-statistic values were the most frequently used indices for evaluating discrimination performance, ranging between 0.72 and 0.89. with the Hosmer-Lemeshow test being the most frequently employed method.

Six of these reporting on six internal validations. The development and validation of the prediction models was facilitated by the utilisation of machine learning algorithms.

### Model validation

Six of the aforementioned subjects reported a total of six internal validations. The development and validation of the prediction models was facilitated by the utilisation of machine learning algorithms.

### Results of quality assessment

Following comprehensive evaluation using the PROBAST checklist, all the studies were categorised as having a high overall risk of bias. The risk of bias and applicability assessments for all the studies are summarised in Table 3. Six studies were adjudged to have a high risk of bias, indicating methodological concerns in the development or validation processes of the models (18, 19, 24–27).

**Table 3:** PROBAST results of the included studies.

Author/year	ROB			Applicability			Overall		
	Participants	Predictors	Outcome	Analysis	Participants	Predictors	Outcome	ROB	Applicability
Onaitis et al. (24)	+	+	−	+	+	+	−	−	−
Zhang. (26)	+	+	+	−	+	+	+	−	+
Zhang et al. (25)	−	+	−	−	−	+	−	−	−
Chen et al. (19)	−	+	+	−	−	+	+	−	−
Niu. (27)	−	+	−	−	−	+	−	−	−
Tong et al. (18)	−	+	−	−	−	+	−	−	−

PROBAST, prediction model risk of bias assessment tool; ROB, risk of bias.

+, indicates low ROB/low concern regarding applicability; −, indicates high ROB/high concern regarding application;.

A total of four studies demonstrated a high risk of bias in the participant domain, which was primarily attributable to the use of inappropriate data sources (18, 19, 25, 27). A total of four studies were identified as being at high risk of bias in the outcome domain. This was primarily attributable to the absence of a blinded assessment of outcomes and predictors (18, 24, 25, 27). A total of four studies confronted the issue of inadequate sample size. More specifically, the number of events per independent variable fell below 20 cases in the model development studies and less than 100 cases in the model validation studies (18, 25–27). In four studies, the partial conversion of continuous variables into categorical variables was employed (19, 25–27). A total of four studies exhibited missing data that was not adequately addressed (18, 19, 26, 27). In the studies under review, the selection of predictors was not based on univariate analysis (18, 19, 27). Moreover, it is evident that the intricacies inherent within the data were not given due consideration in two studies (18, 27). A final study did not consider the overfitting, underfitting and optimism performances of the models in a comprehensive manner (26). In terms of the applicability risk assessment, one study was categorised as low-risk (26), and five were categorised as high-risk (18, 19, 24, 25, 27). With regard to the participant dimension, one study exhibited a high risk due to its recruitment of participants aged 60 and above (25).

### Meta-analysis of validation models

The predictive performance of the eligible models was synthesised through meta-analysis. In view of the absence of adequate data concerning model characteristics and performance metrics in the preliminary search, a mere six studies were considered to be suitable for quantitative synthesis of discriminative ability. In instances where 95% confidence intervals (CI) for the Area Under the Curve (AUC) were not explicitly provided, the Hanley and McNeil method was employed to estimate the standard error from the reported AUC value and the corresponding sample size. This was conducted with the objective of facilitating subsequent pooled analysis (28, 29).

The overall predictive performance of the six validation models was quantified using a random-effects model to account for anticipated methodological and clinical variability.

**Figure 3:**
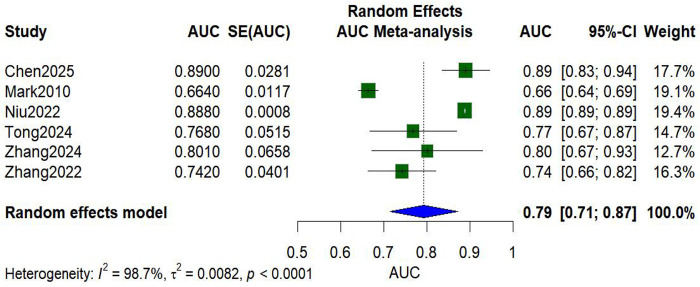
Forest plot of the random effects meta-analysis of pooled AUC estimates for six validation models.

The meta-analysis of all six studies yielded a pooled AUC of 0.79 (95% CI: 0.71–0.87). This value indicates a satisfactory overall diagnostic value of the pooled models. However, this overall synthesis demonstrated a high degree of statistical heterogeneity, with an *I*^2^ value of 98.70% (*p* < 0.001), suggesting that the observed variability in predictive performance is predominantly due to true differences across studies rather than chance (Figure 3).

**Figure 4:**
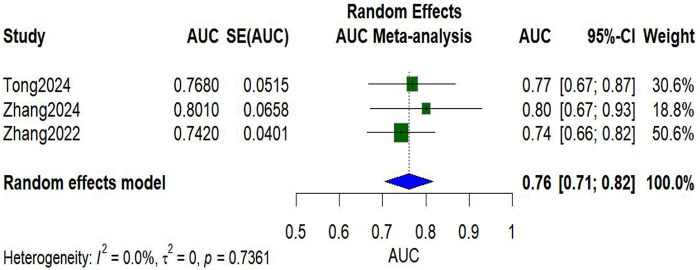
Forest plot of the random-effects meta-analysis of the pooled AUC estimates for the atrial fibrillation outcome from three validated models.

In order to address this high heterogeneity, a subgroup analysis was performed based on the specific definition of the outcome. For the subset of models that explicitly employed atrial fibrillation as the defined clinical outcome, the pooled area under the curve (AUC) was 0.76 (95% CI: 0.71–0.82) (see Figure 4). A subsequent subgroup analysis revealed a minimal degree of heterogeneity, as evidenced by an *I*^2^ value of 0.0% (*p* = 0.736). This finding suggests that when the outcome is uniformly defined as atrial fibrillation, the models exhibit consistent predictive accuracy, lending greater confidence to this specific estimate.

In order to evaluate the stability and reliability of the meta-analysis results, a leave-one-out sensitivity analysis was conducted. This process entailed the sequential exclusion of each individual study, with the summary AUC being recalculated for each iteration.

**Figure 5:**
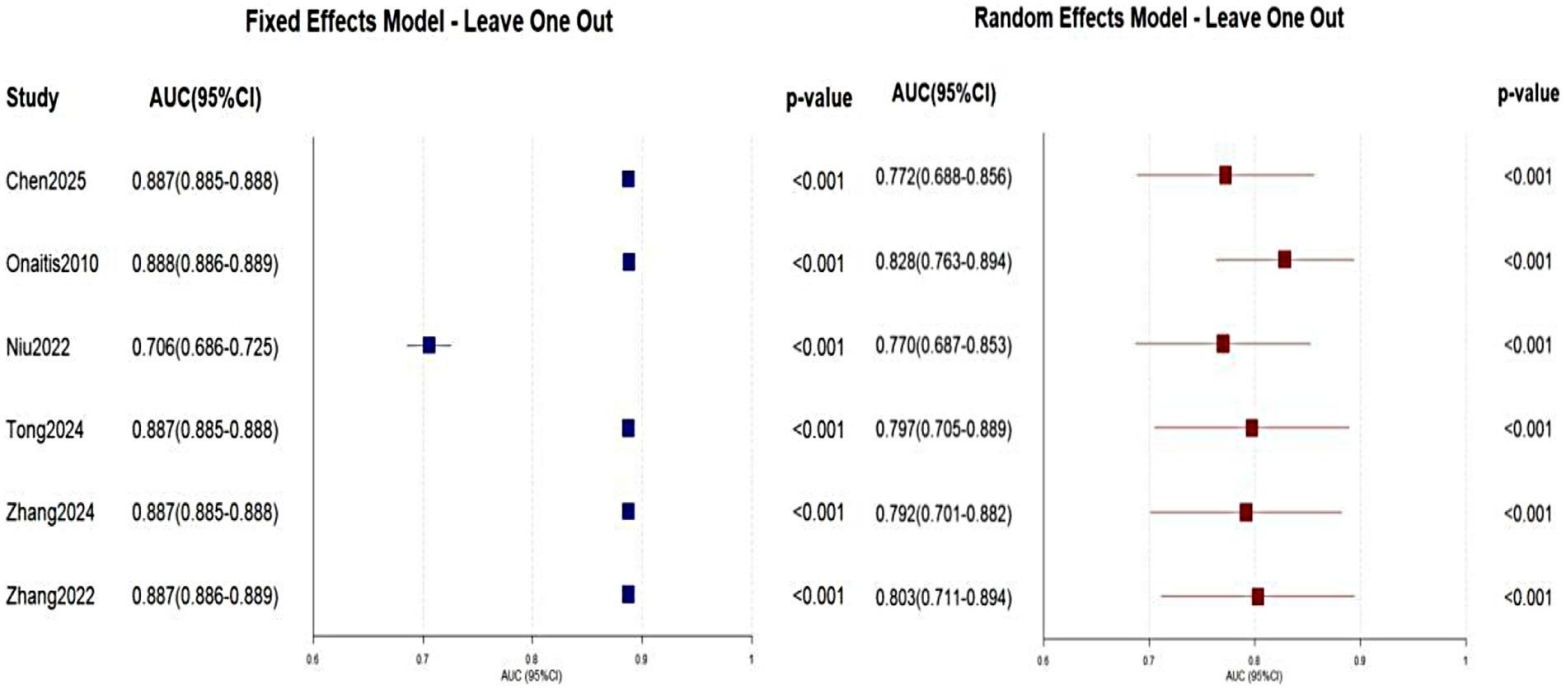
Sensitivity analysis using the leave-one-out model.

The random-effects model demonstrated the robustness and stability of the overall results (Figure 5). It was established that all recalculated Area Under the Curve (AUC) values remained above 0.75, ranging from 0.770 to 0.828. This consistently good diagnostic value serves to reinforce the reliability of the pooled AUC derived under the more conservative random-effects assumption.

Conversely, the sensitivity analysis undertaken under the fixed-effects model indicated a substantial reliance on a single study (see Figure 5). The majority of studies were excluded from the analysis, resulting in a stable area under the curve (AUC) range of 0.887–0.888. However, the exclusion of the study by Niu (27) led to a notable decrease in the pooled AUC to 0.706 (95% CI: 0.686–0.725). This substantial impact suggests that the Niu (27) study holds a disproportionately high weight or deviates significantly in performance within the fixed-effects calculation. This highlights the importance of using the random-effects model, which better accounts for inter-study variance, to report the final, most reliable estimate of predictive performance.

In summary, the meta-analysis confirms that, despite considerable heterogeneity in the overall pool, the included prediction models possess good pooled diagnostic performance (AUC: 0.79). The robustness of the results under the random-effects sensitivity analysis further supports the reliability of this finding.

## Discussion

This systematic review and meta-analysis synthesised the predictive performance of models for POAF risk in lung cancer patients, consolidating evidence on model discrimination and highlighting critical areas for future development.

### Predictive performance and clinical actionability

The meta-analysis of six studies assessing risk prediction models for postoperative atrial fibrillation (POAF) in lung cancer patients revealed a pooled Area Under the Curve (AUC) of 0.79 (95% CI: 0.71–0.87), suggesting a generally satisfactory discriminative ability. This is consistent with the findings from Onaitis (24) and Zhang (26), who reported AUC values ranging from 0.66 to 0.85 in their models. Despite this, the AUC < 0.80 threshold raises concerns about the clinical utility of these models (30). Ideal models for high-morbidity complications such as POAF should ideally have AUC > 0.80, reflecting high discrimination capabilities, which current models fail to achieve. The moderate AUC values indicate that while existing models can identify high-risk patients, they are not yet reliable enough for clinical decision-making on an individual level (31). This gap is crucial, particularly in stratifying patients for prophylactic interventions or intensive monitoring post-surgery.

### Detailed analysis of common predictive factors for POAF

Age, gender, and pre-existing cardiovascular conditions are the most frequently identified predictors of POAF in lung cancer patients. Older age is consistently linked to higher POAF risk (19, 25, 27), reflecting age-related physiological changes and comorbidities such as hypertension and coronary artery disease. Similarly, male gender is a significant risk factor, potentially due to the higher prevalence of cardiovascular diseases and hormonal differences that protect women (24, 25). Additionally, a history of cardiovascular disease greatly contributes to the likelihood of developing POAF, as these conditions cause structural and electrical alterations in the heart that predispose patients to arrhythmias (19, 25, 26).

Surgical factors also play a critical role in POAF risk. More invasive procedures like pneumonectomy and lobectomy, as well as thoracotomy compared to minimally invasive approaches like VATS, are associated with higher rates of POAF due to greater surgical trauma, longer recovery times, and increased postoperative complications (19, 27). Additionally, preoperative electrolyte imbalances—especially hypokalemia and hypohemoglobinemia—are key predictors of POAF, as these imbalances can impair myocardial function and contribute to arrhythmogenesis (18, 27).

For future prediction models, it is crucial to incorporate both static preoperative factors and dynamic perioperative factors to improve the accuracy of POAF risk stratification. By developing more comprehensive, multifactorial models, clinicians can better identify high-risk patients and apply timely interventions, ultimately improving patient outcomes post-surgery.

### The impact of methodological heterogeneity

The high degree of heterogeneity observed in the meta-analysis (*I*^2^ = 98.7%) reflects fundamental inconsistencies in research methodologies. This variability is not solely statistical but stems from several methodological differences across studies, including the definition of outcomes, surgical approaches, and risk factor selection. For example, while some models employed logistic regression (18, 24–27), others used risk score models (19). The inconsistency in outcome definitions significantly contributed to this heterogeneity.

Moreover, the inclusion of a mix of single-center and multi-center studies, as seen in Chen et al. (19) and Tong et al. (18), introduces further bias and affects the external validity of the models. The Niu (27) study had a disproportionately high impact on the overall model performance, as shown by the sensitivity analysis, which further underscores the necessity of multi-center external validation to ensure robustness

The majority of models were derived from single-center cohorts, which engenders the risk of optimistic bias and suboptimal external performance. The substantial decline in the pooled AUC upon excluding the Niu (27) study in the fixed-effects model underscores the impact of single-study characteristics on the skewing of results, thereby reinforcing the efficacy of the more conservative random-effects approach.

The following discussion will address the clinical implications and future directions.

In order to bridge the current gap between model development and clinical implementation, it is essential that future development studies incorporate robust, independent external validation cohorts. Furthermore, researchers must give priority to the reporting of model calibration, a component which is often overlooked but is essential for clinical utility. The development of future models should explore the integration of novel biological, electrophysiological and procedural data, such as preoperative inflammatory markers, atrial strain parameters measured by echocardiography, and intraoperative variables, with a view to improving discriminative power. Moving beyond static preoperative scores, the development of dynamic prediction models that incorporate variables collected immediately post-surgery may provide more accurate, real-time risk stratification during the critical initial 48 h.

### Strengths and limitations

A significant strength of this review lies in its rigorous quantitative approach, which incorporates the Hanley and McNeil method to maximise data inclusion (28, 29). Furthermore, it conducts comprehensive sensitivity and subgroup analyses to interpret high heterogeneity.

Nevertheless, it is important to note that there are several limitations that require careful consideration. Firstly, the limited number of studies (*n* = 6) that are eligible for quantitative synthesis reduces statistical power and constrains the generalisability of the pooled estimate. Secondly, a considerable degree of methodological heterogeneity was observed among the included studies (*I*^2^ = 98.7%). Although subgroup analysis indicated that uniform outcome definitions can reduce this variability (*I*^2^ = 0.0%), inconsistencies in variable selection, predictor management, and modelling strategies persist as a source of uncertainty. Thirdly, the preponderance of studies 5/6 employed a retrospective research design. It is evident that such designs are inherently susceptible to selection bias and unmeasured confounding. Consequently, this may result in the estimation of overly optimistic performance measures when compared to prospective validation. Fourthly, the study populations were almost exclusively Chinese, with five out of six studies conducted exclusively or predominantly in Chinese populations. Given the potential for ethnic and genetic variations in susceptibility to atrial fibrillation, as well as differences in surgical practice and healthcare delivery systems, the applicability of these models to other ethnic populations remains undetermined. Furthermore, the reliance on reported Area Under the Curve (AUC) values precluded a more profound meta-analysis of individual predictor weights. In order to facilitate advancement in this field, it is recommended that future investigations place priority on the execution of well-designed prospective cohort studies, adherence to standardised reporting guidelines, and the undertaking of rigorous external validation across a range of diverse geographic and ethnic settings.

## Conclusion

In conclusion, prediction models for POAF in lung cancer patients demonstrate good pooled discriminative performance (AUC 0.79). However, the presence of pervasive methodological flaws, particularly elevated heterogeneity and an absence of rigorous external validation and standardized outcome definitions, currently hinders their clinical adoption as actionable decision-making tools. It is imperative that future research adopts standardised reporting methodologies, mandates external validation processes, and incorporates novel, dynamic predictors to develop models that possess the requisite accuracy and reliability for routine surgical care.

## Data Availability

The original contributions presented in the study are included in the article/Supplementary Material, further inquiries can be directed to the corresponding authors.
